# How Good Is the Machine at the Imitation Game? On Stylistic Characteristics of AI-Generated Images

**DOI:** 10.3390/jimaging11120429

**Published:** 2025-12-02

**Authors:** Adrien Deliège, Jeanne Marlot, Marc Van Droogenbroeck, Maria Giulia Dondero

**Affiliations:** 1 F.R.S.-FNRS, Rue d’Egmont 5, 1000 Brussels, Belgium; mariagiulia.dondero@uliege.be; 2 Department of Electrical Engineering and Computer Science, Montefiore Institute, Faculty of Applied Sciences, University of Liège, 4000 Liège, Belgium; m.vandroogenbroeck@uliege.be; 3 Department of Romance Languages and Literatures, Faculty of Philosophy and Letters, University of Liège, 4000 Liège, Belgium; j.marlot@uliege.be

**Keywords:** text-to-image generation, Midjourney, artistic style, art history, visual semiotics, stylistic fidelity, expert evaluation

## Abstract

Text-to-image generative models can be used to imitate historical artistic styles, but their effectiveness in doing so remains unclear. In this work, we propose an evaluation framework that leverages expert knowledge from art history and visual semiotics and combines it with quantitative analysis to assess stylistic fidelity. Three experts rated both historical artwork production and images generated with Midjourney v6 for five major movements (Abstract Art, Cubism, Expressionism, Impressionism, Surrealism) and ten associated painters (male and female pairs), using nine visual criteria grounded in Greimas’s plastic categories and Wölfflin’s stylistic oppositions. Ratings were expressed as 95% intervals on continuous 0–100 scales and compared using our Relative Ratings Map (RRMap), which summarizes relative shifts, relative dispersion, and distributional overlap (via the Bhattacharyya coefficient). They were also discretized in four quality ratings (bad, stereotype, fair, excellent). The results show strong inter-expert variability and more moderate intra-expert effects tied to movements, criteria, criterion groups and modalities. Experts tend to agree that the model sometimes aligns with historical trends but also sometimes produces stereotyped versions of a movement or painter, or even completely missed its target, although no unanimous consensus emerges. We conclude that evaluating generative models requires both expert-driven interpretation and quantitative tools, and that stylistic fidelity is hard to quantify even with a rigorous framework.

## 1. Introduction

### 1.1. Context and Problem Statement

In recent years, text-to-image generative models such as DALL·E [1], Midjourney [2], and Stable Diffusion [3] have seen a remarkable rise in visual production. In 2022, Jason Michael Allen won the “Digital Arts/Digitally Manipulated Photography” category of the Colorado State Fair with *Space Opera Theater* [4], an image produced using Midjourney. The following year, the Hollywood strikes highlighted growing concerns across the creative industries about technologies that could replace scriptwriters, graphic designers, or actors [5]. In 2025, the debate continued when the virtual actress Tilly Norwood, generated by artificial intelligence (AI), was presented at the Zürich Film Festival [6]. Although this expanding presence of AI in creative and artistic fields is technically impressive, it remains largely unexplored with respect to the models’ ability to reproduce the established artistic styles that shape our visual culture or to reflect the diversity of works that constitute art history. The present study therefore asks a simple but fundamental question:

Which parts of art history do text-to-image generative models reproduce?

### 1.2. State of the Question in Art History Communities

Art history has integrated the question of AI through several lines of research: the authorship of works resulting from human–machine collaborations; the artistic practices enabled by these tools; the transformations they bring to the reception of artworks; and their impact on the teaching of the visual arts. As examples, one can mention the collective volume published by the Centre Pompidou, *L’Art au temps de l’IA. Générer, critiquer, créer* (in English: *Art in the Age of AI: Generating, Critiquing, Creating*) [7], as well as the *TraAM Arts plastiques 2024–2025* project [8], which focuses more specifically on the pedagogical integration of these issues. However, despite this growing body of research, very few studies have examined the *stylistic characteristics* reproduced by image-generating AIs, even among those focusing on style.

In a survey of professional American illustrators, Porquet et al. [9] showed that these models do not truly reproduce artistic styles, as they operate under a different conception of “style”. For illustrators, style is a dynamic generative principle embedded in a context; for AI models, it seems reduced to texture or a set of frozen, decontextualized statistical patterns. This reduction follows extractivist logics already present in the creative industries, devaluing artistic labor by allowing clients to generate images from a simple textual description. As Meyer [10] summarizes, “thus ‘style’ ceases to be a historical category and becomes a pattern of visual information to be extracted and monetized”.

In visual semiotics, Manovich [11], for his part, observes that these systems tend to produce a default “house style,” a standardized visual identity that appears whenever no specific instruction is given. According to Dondero [12], however, this explanation is too simplistic: the style of AI is not defined by opposition, as in art history, but rather by subtraction. By testing the reproduction of institutionalized artistic styles, one can reveal, by contrast, the “hand of the machine,” that is, what resists imitation and constitutes the AI’s own signature.

More recently, the works of D’Armenio et al. [13,14,15] and Deliège et al. [16] have sought to fill this gap by analyzing how Midjourney, DALL·E, and Stable Diffusion translate textual prompts into figurative and plastic compositions. The originality of their research lies not in generating aesthetic images, but in granting them scientific status by treating prompts as instruments of exploration, i.e., tools for probing regions of a database capable of producing multiple visual possibilities.

Yet, to our knowledge, no methods from the art history community currently allow for the *quantitative* measurement of how faithfully and how diversely generative models reproduce the pictorial genres, movements, and artistic styles of art history. This constitutes the core research question of the present work.

### 1.3. State of the Question in Computer Vision Communities

On the side of “technical sciences”, research on artistic style within computer vision mostly revolves around style representation and transfer, style or author attribution for artworks, and studies on cultural biases and stereotypes in text-to-image generative models.

Neural style transfer introduced a separation of content and style using first- and second-order statistics of convolutional neural network features [17,18]. Subsequent work broadened the topic with higher-order statistics [19] and generalization to several popular model architectures [20]. The community has also emphasized the need for quantitative evaluation beyond visual inspection. ArtFID proposes a stylization quality metric aligned with human judgments for benchmarking [21] neural style transfer. In parallel, ArtScore learns a measure of “artness,” estimating how much an image resembles fine art rather than photography [22]. Together, these efforts motivate criterion-based evaluations of style.

Computer vision has also considered style as a classification problem on historical corpora. For example, Ugail et al. [23] combine deep features and classifiers for authentication in the case of Raphael. Complementary works have outlined oppositions between computer vision and art history, highlighting the issues of treating artworks as ordinary images [24] and asking for better methodological bridges that respect art-historical nuances while enabling quantitative analysis.

The growing literature shows that text-to-image generative models can both reflect and amplify socio-cultural biases present in their training data. A recent survey [25] shows that most works focus on biases related to gender, skin tone, and geo-culture. It also reveals discrepancies in the definition, evaluation, and mitigation strategies of these works. Empirical studies find amplification of gender stereotypes in work roles when comparing Stable Diffusion outputs to training distributions [26]. At inference time, mitigation frameworks such as Fair Diffusion [27] allow bias attenuation based on human instructions, and Fair Mapping [28] based on embedding correction in a debiased space by a linear network. While these works quantify social stereotypes, they typically do not address stylistic fidelity as defined by art history experts, which is precisely the gap addressed in the present study.

### 1.4. Our Contributions

Concretely, we propose a rating protocol aimed at assessing how faithfully text-to-image generative models reproduce established artistic styles. In addition, we apply our protocol to evaluate the ability of *Midjourney v6* to reproduce, typify, or distort the stylistic traits associated with five major art movements and five pairs of painters representative of these movements (one well-known male artist and one historically less represented female artist).

The particular strength of our approach is that we combine expert judgment with quantitative analyses. Three specialists with an academic knowledge of art history and visual semiotics evaluated both real artwork production and AI-generated images using nine visual criteria operationalized from the plastic categories (topological, eidetic, and chromatic) defined by Greimas et al. [29] and from the stylistic classical-baroque oppositions established by Wölfflin [30,31]. Each criterion is expressed as a pair of oppositional poles defining a continuous 0–100 scale (*e.g.*, *centered forms [0] vs. edge-deployed forms [100]*).

For each movement and each criterion, experts performed six ratings: the real artworks of the movement, those of the selected male and female painters, and the corresponding images generated by Midjourney using prompts referring to the same movement and painters. Ratings were expressed as intervals encompassing approximately 95% of the images evaluated, thus reflecting both dominant stylistic features and intrinsic stylistic variability.

To compare these expert evaluations, we introduce a quantitative comparison protocol that computes the relative shift and dispersion of intervals. We also map them on our *Relative Ratings Map* (RRMap) and derive bounds to determine when AI-generated images are faithful imitations or stereotyped versions of the corresponding criteria, giving discrete *quality ratings*. The background color of the RRMap encodes the Bhattacharyya coefficient, seen as a measure of overlap, between the two intervals of interest. We perform statistical tests to assess inter-expert and intra-expert rating variability, overall and per movement, criterion, groups, modalities, for both RRMap ratings and quality ratings.

Our results indicate that experts diverge in their evaluations of AI-generated artworks, both globally and at the level of individual stylistic criteria. While two experts occasionally align in their ratings, no universal consensus emerges, underscoring the subjective nature of stylistic judgment. Within each expert, specific factors such as movement or evaluation criteria suggest that the model reproduces some stylistic patterns while simplifying or stereotyping others. Overall, the study shows both the interpretive variability of human experts and the uneven fidelity with which text-to-image models capture the nuances of art historical style.

The remainder of this article is organized as follows. Section 2 presents our materials and methods, detailing the construction of the image corpus, the evaluation criteria, the expert rating protocol, the rating comparison protocol, the RRMap and its interpretation, the quality ratings derived from it, and the statistical tests. Section 3 reports the results and discussions of our comparative analyses, followed by their summary and additional expert feedback in Section 4. Finally, Section 5 concludes the paper and opens the door to future work.

## 2. Materials and Methods

### 2.1. Materials

Our working material consists of images generated by a text-to-image model, which were produced after making a few choices, are detailed below.

#### 2.1.1. Choice of Artistic Movements

To examine the capacity of text-to-image models to reproduce, stereotype, or fail to capture artistic styles, we selected five of the most prominent movements in art history: **Abstract Art, Cubism, Expressionism, Impressionism, and Surrealism**. These movements were identified by art historians as representative of a substantial portion of historical artistic production, while being sufficiently diverse to allow for meaningful comparative analysis. Moreover, they are general and well-known enough that text-to-image models are likely to have encountered numerous references to them during training, making it plausible to assume that the models have developed internal representations of these movements. In contrast, lesser-known movements would likely suffer from under-representation in training datasets, potentially leading the models to generate images unrelated to the intended artistic style.

#### 2.1.2. Choice of Painters

To assess whether artistic movements are reducible to the style of specific canonical painters (either by expert human raters or by the generative model itself), we included both widely recognized male painters and less frequently cited but historically significant female painters associated with each movement. The inclusion of female painters serves two purposes: first, to evaluate whether the model captures different stylistic cues from a less famous painter than those related to famous painters; and second, to estimate how much historical gender asymmetries in artistic fame and data representation have been absorbed by the model. The selected pairings were: **Wassily Kandinsky** and **Hilma af Klint** for Abstract Art, **Pablo Picasso** and **Marie Laurencin** for Cubism, **Ernst Ludwig Kirchner** and **Gabriele Münter** for Expressionism, **Claude Monet** and **Mary Cassatt** for Impressionism, and **Joan Miró** and **Dorothea Tanning** for Surrealism.

#### 2.1.3. Choice of a Text-to-Image Generative Model

To offer a deep art-historical perspective on machine-generated imagery rather than to conduct a high-level comparative evaluation across many models, we chose to focus on a single system that emphasizes visual aesthetics and stylistic rendering. For this reason, we selected **Midjourney** [2], which has been previously noted for its aesthetic and stylized output tendencies [13,15]. All images were generated using Midjourney version 6 with default parameters through its Discord-based interface.

#### 2.1.4. Design of Prompts

To minimize interpretative variability and ensure experimental control, we used short and unambiguous prompts designed to isolate core stylistic concepts. For each of the five movements, we used the prompt template **“A <movement> painting.”**, where <movement> corresponds to abstract, cubist, expressionist, impressionist, or surrealist. For each of the ten painters, we used the prompt template **“A painting in the style of <painter>.”**, where <painter> denotes the full name of the artist (e.g., Pablo Picasso). This resulted in a total of fifteen distinct prompts.

##### Choice of Number of Generated Images

To achieve a balance between statistical robustness and the feasibility of expert evaluation, we generated **twenty images per prompt**, yielding a total of 300 images across all prompts, which constitutes our raw working material.

### 2.2. Methods

#### 2.2.1. Preliminaries: Greimas and Wölfflin Frameworks

The rating protocol used in this study relies on nine non-exhaustive parameters designed to characterize the visual composition of images. It combines perspectives from art history and visual semiotics, drawing primarily on the *plastic categories* defined by Greimas et al. [29] and on the formal criteria established by Wölfflin [30,31] to distinguish classical and baroque characteristics. These two authors proposed complementary approaches for describing how visual forms convey meaning independently of representational content. Their respective frameworks, although developed in different historical contexts, converge toward a common goal: understanding how compositional relations organize the perceptual unity of an image.

Greimas’s semiotic theory of plasticity (also known as formal composition) proposes to analyze images through three fundamental categories: *topological*, *eidetic*, and *chromatic*, each defined by oppositional relations. The topological category concerns the spatial arrangement of components in terms of contrasts such as center vs. periphery, left vs. right, or top vs. bottom. The eidetic category pertains to the forms and their contours, distinguishing rectilinear from curvilinear, fragmented from continuous, and parallel from intersecting structures. The chromatic category, more continuous by nature, involves contrasts of color, brightness (light vs. dark), and material effects (smooth vs. rough) that contribute to the overall perception of form. In relation to light, Fontanille [32] introduced the useful distinction between *illumination*, referring to homogeneous lighting, and *brightness* or *glare*, referring to dazzling intensity.

Wölfflin, on the other hand, developed a comparative art-historical method based on a series of stylistic oppositions that characterize classical and baroque visual regimes: linear vs. painterly, planar vs. recessive, closed vs. open form, multiplicity vs. unity, and clarity vs. obscurity. While Greimas focused on local oppositions between plastic features, Wölfflin conceived of the image as an integrated composition, analyzing how light, form, and space cohere into an overall visual order. His distinction between classical and baroque optics reveals how images can oscillate between stability and dynamism, between internal containment and outward expansion.

The rating criteria adopted in this work articulate these two traditions by translating their conceptual oppositions into nine operational criteria that describe the compositional, eidetic, and chromatic organization of an image. Each criterion is expressed as a continuum between two poles (e.g., centered vs. edge-deployed forms), emphasizing that visual properties rarely occur in absolute terms but rather occupy intermediate positions within a continuous perceptual spectrum.

#### 2.2.2. Design of Stylistic Evaluation Criteria

To assess the stylistic properties of the real paintings and of the generated images, we designed a set of **nine visual criteria grounded in classical art-historical and semiotic frameworks**. Following the plastic categories defined by Greimas and the stylistic oppositions formulated by Wölfflin, our criteria are split into three groups, each related to a main dimension of analysis: topological, eidetic, and chromatic. Each criterion represents a continuum between two poles, allowing fine-grained evaluation of compositional tendencies in both human paintings and generated images.

##### Topological Criteria

The first group, consisting of three topological criteria, concerns the spatial organization of the composition. The criterion **centered forms vs. edge-deployed forms** distinguishes compositions according to whether their internal dynamics are centripetal or centrifugal. Following Wölfflin’s analysis, centered compositions are those in which all represented figures fit entirely within the frame, producing a balanced image characteristic of a classical painting, while forms extending to the edges correspond to baroque-like compositions, where figures exceed the frame or direct the viewer’s attention outward through diagonals or asymmetries that suggest imbalance. Two additional topological criteria capture the internal arrangement of visual elements: **left–right symmetry vs. asymmetry** and **top–bottom symmetry vs. asymmetry**. In human artistic practice, asymmetry is often exploited to increase the dramatic effect. Picasso, for instance, uses imbalance to create a sense of strangeness. On the other hand, in portraits, artists generally favor symmetrical figure–ground relations.

##### Eidetic Criteria

The second group, consisting of three eidetic criteria, pertains to the treatment of form and line: **sharp vs. blurred images**, **parallel vs. intersecting lines**, and **dense vs. sparse rhythmic structures**. The sharpness criterion, introduced within the more general “texture” umbrella by the Groupe μ [33], concerns the clarity of contours and their overall resolution. Historically, blur emerged in photography [34,35,36] as both a technical limitation and later as an artistic device to evoke painterly effects, a notion also present in earlier painting practices such as Leonardo da Vinci’s *sfumato* and the blurred zones in Francis Bacon’s paintings [37,38]. The distinction between parallel and intersecting lines derives from Greimas’s eidetic category and Wölfflin’s classical/baroque opposition. Classical compositions favor parallelism, whereas baroque compositions feature intersecting diagonals that generate a sense of chaos or mass. The rhythm of lines further differentiates images by their visual density: dense rhythms, as in Futurist works like Giacomo Balla’s *Little Girl Running on the Balcony* (1912), convey dynamism and vibration, while sparse rhythms, as in *Numbers in Love* (1920) or *The Car Has Passed* (1913), produce a calmer, more open spatial texture. Let us note that in our context, “lines” refer to structural features of composition rather than individual brushstrokes.

##### Chromatic Criteria

The third group, consisting of three chromatic criteria, addresses color, light, and texture. The first criterion, **saturated vs. desaturated colors**, measures the chromatic intensity of hues. The second, **uniform vs. focalized lighting**, follows Fontanille’s distinction [32] between *illumination* and *brightness*, echoing Wölfflin’s analyses of classical and baroque light. When light serves the form (i.e., reveals shapes clearly), the composition aligns with classical aesthetics; when light obscures the form through shadow or glare, it belongs to a baroque register. The third, **painterly vs. photographic texture**, concerns the visual materiality of the image. Drawing on Fontanille’s concepts of *substrate* (the receptive surface) and *application* (applying the substance), this criterion interprets texture as the result of an act of inscription. In painting, the *application* manifests through brush movement, while in photography it depends on light capture and printing processes.

#### 2.2.3. Design of the Expert Rating Protocol

To quantify the agreement between the generated images and art-historical characteristics, **three experts in art history and visual semiotics** independently rated each artistic movement and **modality (movement, male painter, female painter)** for each possible **origin (real paintings or generated images)** and criterion, using the continuous scales defined above. For each expert, this amounts to evaluating 5 movements × 3 modalities × 2 origins × 9 criteria, yielding 270 ratings per expert and thus 810 ratings in total.

Each expert conducted the rating one movement at a time. For a given movement, the expert received the full set of images generated for the 3 modalities of interest, i.e., produced from the prompts "A <movement> painting." and “A painting in the style of <painter>.” for the two painters associated with that movement. The twenty images corresponding to each modality were presented together as **a single composite “meta-image” made of twenty sub-images**, allowing the experts to consider the overall distribution of stylistic characteristics. Preliminary trials with our experts had shown that rating images individually rather than in groups produced largely inconsistent results and hindered any kind of meaningful comparison (according to the experts); the grouped presentation provided a much more suitable rating protocol.

Then, given a movement and the three meta-images, the experts proceeded one criterion at a time, viewing and **adjusting six corresponding evaluations within the same rating sheet** (3 per modality × 2 per origin). This shared setup facilitated relative comparisons across modalities and origins. Early pilot sessions with fully blind, independent ratings revealed the absence of an absolute rating scale across movements and origins, thus underlining that reliable judgments could only be obtained through relative comparisons within the movement.

**Figure 1:**
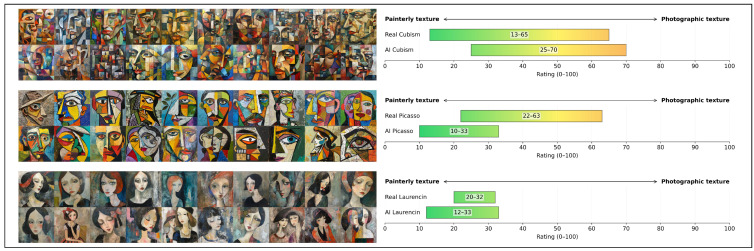
**Example of the rating protocol** for Cubism, on a single rating sheet. For each meta-image on the left, obtained from the prompts (top) “A Cubist painting”, (middle) “A painting in the style of Pablo Picasso”, and (bottom) “A painting in the style of Marie Laurencin”, the right-hand panels show the corresponding expert rating intervals on a 0-100 scale for the criterion “Painterly texture vs. Photographic texture”, for both historical (real) paintings and AI-generated images.

Ratings were expressed as **intervals on a 0–100 continuous scale**, rather than single values, to reflect the natural variability of stylistic traits within both historical paintings and generated images. Experts were instructed, for the historical reference, to specify an interval **encompassing approximately 95%** of the known artworks representative of that modality, allowing for legitimate exceptions and avoiding unnecessary stretching due to marginal cases. Similarly, for the generated images, experts defined an interval that included roughly 95% (i.e., 19 out of 20) of the presented images, with a comparable tolerance for rare outliers. We set up this interval-based method because **we assume that each criterion follows an approximately normal (Gaussian) distribution**. We motivate this assumption by two reasons: Requesting a 95% interval enabled non-technical experts to indicate both a mean tendency and its associated dispersion intuitively, without requiring any statistical knowledge; and if an expert was able to rate many batches of many images, then the Central Limit Theorem asserts that the means of the ratings of the batches would be normally distributed. We bypass this tedious work by assuming that the experts are knowledgeable enough to be able to provide approximate distributions directly. An example of a rating sheet is shown in Figure 1.

Let us note that the experts did not see any particular selection of historical paintings. Instead, they were asked to rate them from their own knowledge. On one hand, the historical production is too large to be sampled efficiently. On the other hand, experts are assumed (and confirmed being) able to rate historical productions globally with our intervals. Still, they were also allowed to use any resource of their choice to look for historical paintings if deemed necessary (for instance for lesser known artists). Furthermore, our early tests revealed that a blind rating of images (without specifying their origin, historical or generated) would be useless since it is utterly easy for the experts (and even for non-experts) to determine which image is AI-generated. This is likely due to the simple fact that known paintings are easily identifiable, in particular by experts, and to the existence of some “hand of the machine” as mentioned previously by Dondero [12] that confers generated images a style that is easily perceived by human raters.

#### 2.2.4. Design of the Ratings Comparison Protocol and the RRMap

To compare two ratings (e.g., one for a real modality and its generated counterpart) each defined by confidence intervals $I_{1}=\left[a_{1},b_{1}\right]$ and $I_{2}=\left[a_{2},b_{2}\right]$, we proceed as follows. Each interval is assumed to represent a 95% confidence range of an underlying normal distribution. Accordingly, we compute the **means** and **standard deviations**:

(1) $$m_{i}=\frac{a_{i}+b_{i}}{2},\;s_{i}=\frac{b_{i}-a_{i}}{2\;z_{0.975}},$$

where $z_{0.975}=1.96$ corresponds to the 97.5th percentile of the standard normal distribution, delimiting the central 95% probability interval. This yields the reparameterizations $I_{1}↔N\left(m_{1},s_{1}\right)$ and $I_{2}↔N\left(m_{2},s_{2}\right)$.

Taking the first distribution (e.g., the real modality) as the reference and the second (e.g., the generated modality) as the one evaluated against it, two quantities summarize their relative configuration:

(2) $$\Delta =\frac{m_{2}-m_{1}}{s_{1}},\;R=\frac{s_{2}}{s_{1}}.$$

The **relative shift**, Δ, captures how the two distributions are aligned in terms of their centers, expressed in units of the reference spread, while *R* **expresses the relative dispersion**. Each comparison thus yields one point (Δ,R) in a two-dimensional space. The normalization of mean differences by $s_{1}$ to compute Δ ensures that all comparisons are expressed in a common scale-independent coordinate system across our analyses. For example, that means comparing the ratings of an expert $I_{1}=\left[30,40\right]$ and $I_{2}=\left[35,45\right]$ is equivalent to comparing the ratings of another expert $I_{3}=\left[60,80\right]$ and $I_{4}=\left[70,90\right]$, in the sense that they both agree on a same level of relative correspondence between their intervals, while their absolute values do not matter and might be affected by one’s personal judgment. They will thus yield the same point in the RRMap. We can cover the case of scale differences as well. For instance, if an expert compares intervals [50, 60] and [45, 65], and if another compares intervals [30, 60] and [15, 75], then again both experts will yield the same point in the RRMap.

To further quantify their **overall similarity**, we compute the **Bhattacharyya coefficient (BC)** [39] between the two normal distributions:

(3) $$BC\left(N_{1},N_{2}\right)=\sqrt{\frac{2s_{1}s_{2}}{s_{1}^{2}+s_{2}^{2}}}\exp\;\left[-\frac{{\left(m_{2}-m_{1}\right)}^{2}}{4(s_{1}^{2}+s_{2}^{2})}\right].$$

This well-known coefficient, ranging from 0 (no overlap) to 1 (identical distributions), can be seen as a measure of the **amount of overlap** between the two Gaussian curves. Conceptually, the procedure is equivalent to normalizing the reference distribution to a standard normal N(0,1), and comparing the second distribution against it (normalized by the parameters of the reference distribution).

To interpret consistently the results of our comparisons, we provide a visualization tool that we call our **Relative Rating Map (RRMap)**. It is the two-dimensional plot displaying (Δ,R) for any subset of movements, criteria, modalities, and experts, on top of a background colored according to corresponding BC values. Our RRMap can be seen as a complementary tool to existing stylistic evaluation metrics such as ArtFID and ArtScore. They all aim at quantifying stylistic fidelity in one way or another. The main difference is that our RRMap explicitly encodes and visualizes an expert (or any rater) evaluation that compares, according to a criterion of interest, a reference production with a query production, with an interpretable meaning (relative shifts and dispersions according to criteria of interest). The other metrics simply provide an expert-free number, useful for benchmarking style transfer capabilities of models but useless for analyzing in detail (e.g., for a specific criterion) the strengths and weaknesses of said models beyond a general score. Our RRMap also aims at characterizing distributions of reference and query productions, not just single specific images, which gives insights on more general trends that might be more useful to grasp the big picture of stylistic fidelity in image generation models.

#### 2.2.5. Choice of Statistical Tests for Experts and Category Effects in the RRMap

To evaluate spatial distribution differences in the RRMap, we use nonparametric **energy-based tests** [40,41,42]. These tests quantify discrepancies between multivariate distributions through pairwise Euclidean distances and are sensitive to variations in overall shape, as well as in location and spread. For each test, *p*-values were calculated from 5000 permutation distributions to generate reliable null distributions. We interpret *p*-values as indicators of evidence rather than hard binary thresholds. In that spirit, we will qualify as “significant” observations with p<0.05 (rejecting the null hypothesis of identical distributions), but without making assertive claims about successes or failures. We tested the following.

*Expert effect (inter-expert variability)*. To test whether our three experts produced significantly different ratings in the RRMap globally (independently of any specific category among movement, criterion, criterion group, modality), we applied a three-sample energy test under the null hypothesis that all annotators share identical joint distributions. We then conducted pairwise two-sample tests to identify which expert pairs differed in their distributions.

*Category effect per expert (intra-expert variability)*. To examine whether individual experts differentiated among categories (e.g., movement, criterion, criterion group, modality) in the RRMap, we repeated the *k*-sample energy test separately for each expert, with *k* being the distinct number of options per category (5 for movements, 9 for criterion, 3 for criterion group, 3 for modality). Then, we performed pairwise two-sample tests between all pairs of options within a category, again computed independently for each expert, always testing the null hypothesis that distributions are identical.

#### 2.2.6. Design of the Interpretation Protocol of the RRMap

The RRMap features particular areas of interest that characterize how similar two ratings are or how much one is a subset of the other, leading to potential stylistic stereotypes. We provide interpretative cues of the RRMap hereafter.

In the RRMap, horizontal displacement reflects the relative shift: points to the left of the origin (Δ<0) indicate that the criterion was rated lower for the second evaluated modality than for the reference, whereas points to the right (Δ>0) indicate higher ratings. Points distant from the vertical axis (|Δ|≫0) indicate biases or stylistic drifts away from the intended criterion. Vertically, R>1 denotes a broader spread (greater variability) in the evaluated modality, and R<1 denotes a narrower spread (reduced diversity). Points low on the RRMap (R≪1) reflect more stereotyped or homogenized generations, while high points (R≫1) correspond to less controlled or inconsistently captured stylistic traits. **The theoretical “perfect stereotype” would thus lie near the coordinate (0,0)**, being characterized by identical mean but minimal variability. **The point (0,1) represents perfect alignment**, where the evaluated modality reproduces both the central tendency and variability of the reference.

To facilitate the interpretation of the RRMap, several qualitative regions were defined based on expert consensus, corresponding to distinct types of relationships between the ratings compared. Following discussions with our experts, when comparing generated images to historical references, we observed that a tendency toward (stereo)typification could be identified when the interval for the generated images was centered within the reference interval and had less than two thirds of its spread. On the RRMap, this delimits the rectangular **stereotype zone**, corresponding to the region where R<2/3 and −2<Δ<2 (theoretical bounds ±1.96, rounded here for clarity). Within this region, the BC values are upper-bounded at (0,2/3) by approximately 0.96, indicating that, according to our experts, stereotypes are more reliably characterized by Δ and *R* values than by BC values alone.

Similarly, for values of Δ within the same range but with 2/3≤R≤3/2, the generated images can be considered a **fair match** to their historical references for the movement and criterion examined. This rectangular region coincides with BC values that are lower-bounded by about 0.5 (specifically 0.494 at (±1.96,2/3)), although BC values alone are insufficient to fully delineate it, since a large part of the stereotype zone also exceeds that threshold.

To define a region of **excellent match**, expert consensus favored a threshold based directly on BC rather than on rectangular boundaries in the RRMap. A value of BC>0.98 was deemed appropriate and serves as our main operational definition of high fidelity between generated and historical styles. This roughly delimits an ellipsoid shape on the RRMap containing the point (0,1). The rationale was that, with equally centered intervals (i.e., Δ=0), one could tolerate a relative dispersion comprised between 3/4 and 4/3 to qualify for an excellent match. On the other hand, the acceptable range for this ratio shrinks as the (absolute) relative shift increases up to 0.4, which corresponds to allowing an excellent match for equally dispersed intervals shifted by at most 10% of the reference interval spread. This ellipsoid shape indicates that a compromise between relative shift and relative dispersion must be met to qualify for an excellent match, i.e., a larger absolute shift (resp. dispersion) is allowed at the cost of a more faithful dispersion (resp. shift) to ensure the overlap between the intervals remains large enough.

Points falling outside these regions are classified as **bad matches**, their corresponding intervals being either too shifted from the reference or excessively dilated. Both cases indicate the model’s failure to capture the examined stylistic characteristic in a satisfactory way. We did not attempt a more granular subdivision of these cases and collectively refer to them as “bad matches”.

**Figure 2:**
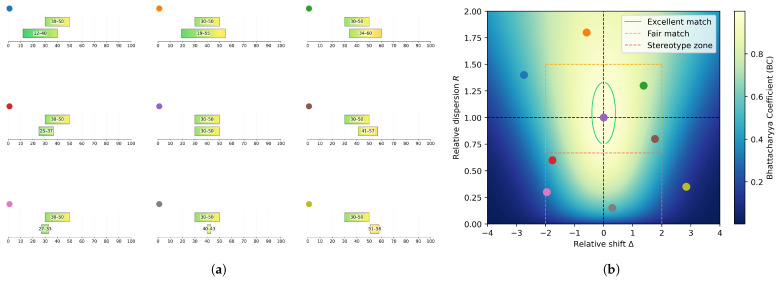
**From ratings to the RRMap**. (**a**) Examples of imaginary ratings, where the reference rating is fixed to the interval [30, 50], and the ratings evaluated against it are either dilated, compressed, and/or shifted, except for the central subplot. (**b**) Representation of these comparisons on the *Relative Ratings Map (RRMap)*, with the Bhattacharyya Coefficient (BC) shown in the background (see Equation (3)). The relative shifts and dispersions refer to how much the ratings are shifted and compacted/stretched compared to the reference ratings on the left panel (see Equations (1) and (2)). The regions of interest, i.e., *stereotype zone*, *fair match*, and *excellent match* are indicated; the remaining region corresponds to *bad matches*.

Figure 2 illustrates the interpretive structure of the RRMap and the main regions of interest discussed above.

#### 2.2.7. Computation of Quality Distributions and Choice of Significance Tests

To assess the correspondence between compared ratings in terms of **quality distribution** (where quality refers to stereotypes, excellent, fair, or bad matches according to the above terminology), natural quantitative metrics consist of the **proportions of points of each quality level**. For brevity, **the quality levels are occasionally abbreviated** as follows: Excellent matches as “**Excel.**”, Fair matches as “**Fair**”, Stereotypes as “**Stereo.**”, and Bad matches as “**Bad**”. For the illustrative case shown in Figure 2, the distribution of points would be: 1/9 Excel., 2/9 Fair, 3/9 Stereo., and 3/9 Bad.

To evaluate differences in the quality levels across experts and categories, we relied on tests based on contingency tables of the frequencies of quality distributions. For all tests, *p*-values were obtained from the corresponding $\chi^{2}$ distributions. We again interpret *p*-values as indicators of evidence rather than as binary decisions, qualifying as “significant” observations those with p<0.05 (rejecting null hypothesis of identical distributions) while avoiding assertive claims. We tested the following.

*Expert effect (inter-expert variability).* To test whether the three experts yielded points distributed in the four rating qualities in comparable proportions, we constructed a 3×4 contingency table (*Expert* × *Rating*) and applied a $\chi^{2}$ test of independence under the null hypothesis that the distribution of ratings is identical across experts. Pairwise 2×4 tables were then analyzed in the same way to identify which expert pairs differed most clearly.

*Category effect per expert (intra-expert variability).* To assess whether each expert differentiated among the possible options of a given category (e.g., movements, criteria, criterion groups, or modalities), we computed separate contingency tables of the form *Category option* × *Rating* for each expert and applied $\chi^{2}$ tests of independence (k×4 tables, with *k* equal to the number of options in the category). Pairwise 2×4 tests were also performed between every pair of options within a category to identify the most salient contrasts.

## 3. Results

### 3.1. Generated Images and Expert Ratings

**Figure 3:**
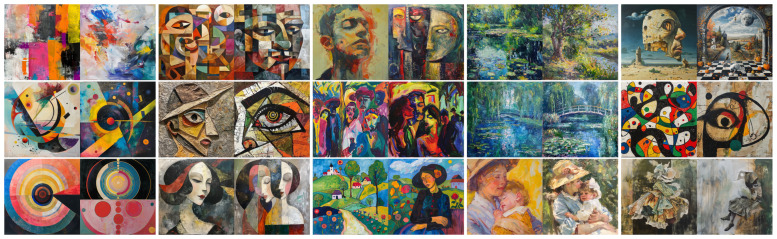
**Examples of images generated for our study.** First row, images generated with the prompt “A <movement> painting.”, showing two images per <movement> listed as *abstract, cubist, expressionist, impressionist, surrealist*. Second row, images generated with the prompt “A painting in the style of <painter>.” with male painters selected per movement, i.e., *Wassily Kandinsky, Pablo Picasso, Ernst Ludwig Kirchner, Claude Monet, Joan Miró*, and third row with female painters, i.e., *Hilma af Klint, Marie Laurencin, Gabriele Münter, Mary Cassatt, Dorothea Tanning*.

Some of the images produced for our study for each movement and painter are shown in Figure 3. All the 300 individual high-resolution images (1024×1024 pixels) are provided in Supplementary Materials File S1, as well as the meta-images used by the experts in the rating sheets.

The 810 ratings provided by our three experts are aggregated in a single CSV file, which is provided in Supplementary Material, with headers following the naming conventions of Section 2: *movement, expert, criterion, criterion group, origin, modality, modality name, low rating, high rating, mean, sigma*. Low and high ratings indicate the lower and upper bounds of the intervals rated by the experts, while means and sigmas (standard deviations) were computed following Equation (1).

### 3.2. Expert Effects (Inter-Expert Variability)

We first examine expert effects, that is, inter-expert variability in the ratings obtained by comparing each historical modality with its generated counterpart, yielding 135 points per expert computed with Equations (2) and (3). We start by assessing expert effects “overall” (without taking categories into account), then we refine the analysis by examining each category separately. As a reminder, our statistical tests are either “global”, i.e., testing the null hypothesis that all distributions considered are identical, or “pairwise”, i.e., testing pairs of distributions for that same null hypothesis. We refer to them as *globally* or *pairwise* in the following.

#### 3.2.1. Overall Ratings

**Figure 4:**
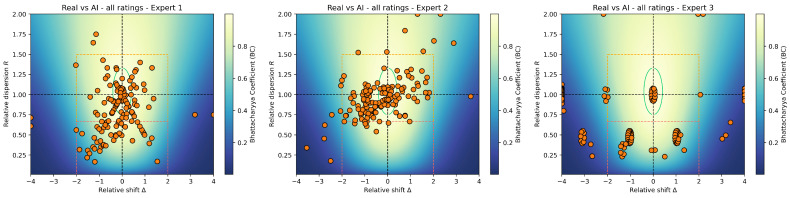
**RRMaps of historical vs. generated modalities** for each expert, giving an “overall” overview of the results, such as quality trends or expert (dis)agreement. We clipped extreme values to keep a useful visualization.

The overall ratings per expert are shown in Figure 4. Visually, it can already be seen that different experts yield different distributions in the RRMap and in the quality regions of interest. Our statistical tests confirm this trend.

*Overall RRMap ratings. Globally*, experts differed significantly in their RRMap rating distributions. *Pairwise* comparisons showed that Expert 3 differed strongly from both Expert 1 and Expert 2 (p<0.001), whereas Experts 1 and 2 were less drastically distinct (p=0.044).

*Overall quality ratings. Globally*, experts also differed significantly in their distribution of the four discrete rating levels. *Pairwise* analyses confirmed significant contrasts between all expert pairs. Interestingly, Expert 3 is the most unconvinced by generated images, with most points Bad (59%) and Stereo. (32%). Expert 1 is relatively balanced across Excel. (26%), Fair (35%), Stereo. (33%). Expert 2 is the most satisfied with generated images quality with respect to historical references, with most points being Fair (61%) or Excel. (21%). The tendency for the model to produce stereotypes is more pronounced for Experts 1 and 3.

*Takeaway.* The four types of tests (RRMap vs. quality × global vs. pairwise) indicate that identical distributions across overall experts ratings are very unlikely, which could indicate significant expert effects in the ratings.

#### 3.2.2. Per Category Ratings

**Figure 5:**
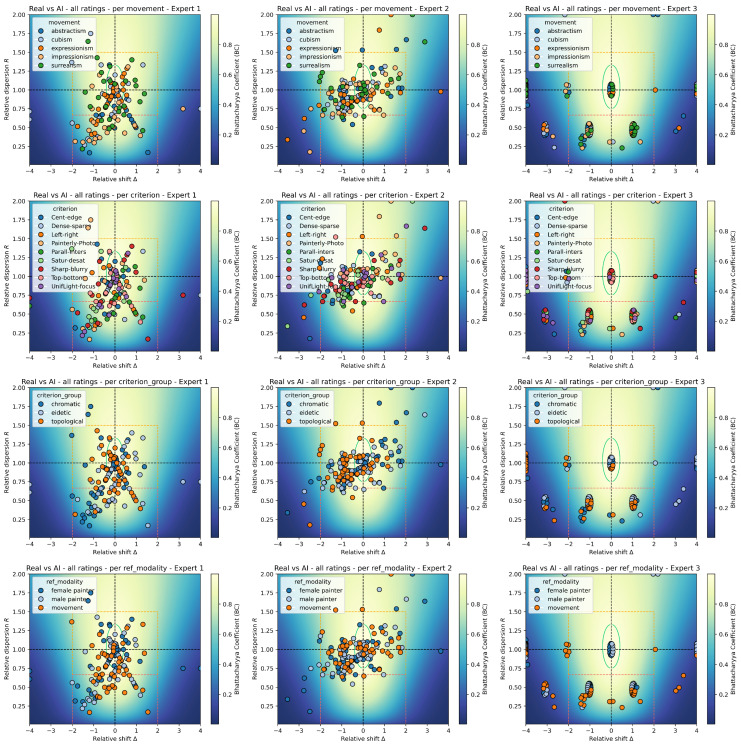
**Per-category (rows) RRMaps of historical vs. generated modalities** for each expert (columns), giving a richer overview of the results, inter-experts and intra-expert.

To refine our analysis and potentially find agreements between experts in some particular cases, we performed the previous tests on subsets of data, corresponding to the various categories of interest (movement, criterion, criterion group, modality). This allows to study expert effects per option within a category. For instance, for the category “movement”, we selected the points corresponding to the option “abstractism” and performed the inter-expert analysis, then repeated the process with the options “cubism”, “expressionism”, etc. The corresponding visualization of the results lies in Figure 5. The analysis carried out in this section thus corresponds to studying expert effects “horizontally” for each row (category) of that Figure, for each option within the categories. In total, this amounts to 5 movements + 9 criteria + 3 criteria groups + 3 modalities = 20 additional analyses (with 4 tests each as previously).

*Per option per category RRMap ratings. Globally*, none of the individual options of any category indicates a potential agreement in the ratings distributions between the experts, excepted the criterion “sharp vs. blurry” for the RRMap ratings (p=0.19). We note, however, that studying one criterion limits us to considering only 15 data points, which increases the likelihood of finding large *p*-values by pure chance. *Pairwise* tests indicate a potential agreement for most RRMap ratings between Expert 1 and Expert 2 (all but the abstract movement, the painterly-photographic criterion, and the topographic and chromatic criterion groups), with pairwise agreements with Expert 3 for the criterion “sharp vs. blurry”.

*Per option per category quality ratings. Globally*, none of the individual options of any category indicates a potential agreement in the ratings distributions between the experts. The *pairwise* quality ratings show significant distribution differences for all options among movements except surrealism (Expert 1 and 2), all criterion groups and all modalities, as well as for 3 criteria (dense-sparse, sharp-blurry, uniform light-focus). The remaining criteria might indicate agreements between Experts 1 and 2, leaning toward a distribution denser in Fair and Excel. qualities, as well as between Experts 1 and 3 for painterly-photo.

*Takeaway.* Experts 1 and 2 might show an agreement on most of the individual (options per category) RRMap ratings and on most of the individual criteria for the quality ratings, in which case the quality of the generated images was deemed mostly high (Fair or Excel). No global consensus seems to be found when selecting subsets of data corresponding to one option in a category.

#### 3.2.3. Further Inter-Experts Analyses

Selecting not one but two options of distinct categories limits our study to only a few points. The “best” (or least worst) case scenario is fixing a criterion group and a modality, in which case 15 points per expert are available. With all the caution required in such a setting, Experts 1 and 2 might consistently agree on all RRMap distributions and most quality distributions (all but when fixing “eidetic—movement” and “chromatic—male painter”). Selecting other pairs of categories yield too few points (e.g., 3 points per expert if movement and criterion are fixed). Therefore, statistical tests are too unreliable to provide meaningful information.

Finally, without looking a distributions of points, we can look at individual points and how they can differ in expert ratings. Backing up the above observations, many points are placed very differently according to the experts. For instance, the point corresponding to abstractism-cent/edges-male painter (Kandinsky) is in the Excel. zone for Expert 1, Fair for Expert 2, and Bad for Expert 3. In terms of quality ratings, the three experts agree only on two points: abstractism-dense/sparse-female painter (H. af Klint) always in the Excel. zone, and surrealism-parall/inters-movement modality always in the Stereo. zone. Among the remaining points, 58 (43% of the points) are placed in three different zones, while partial agreement occurs for 27 points (resp. 15, 33) between Experts 1 and 3 (resp. 2 and 3, 1 and 2).

*Takeaway.* Experts 1 and 2 might show an agreement on subsets of data limited to a particular criterion group and modality, in line with previous observations. Pointwise quality ratings indicate little agreement between the three experts, many complete disagreements, and some partial agreements distributed among expert pairs.

### 3.3. Per Expert Analysis and Category Effects (Intra-Expert Variability)

Given the significant differences between expert ratings, we sharpen our analysis on a per-expert investigation. For each expert, we examine whether the distributions of ratings are tied to category-specific options. Concretely, we study the effect of the movement, the criterion, the criterion group, and the modality. The analysis carried out in this section thus corresponds to studying category effects “vertically” for each column (expert) of Figure 5, *globally* across options within a category (i.e., testing the hypothesis that the options all follow identical distributions) and *pairwise*.

#### 3.3.1. Effect of Movement

*RRMap ratings. Globally*, Experts 1 and 3 show a significant effect due to movement, while our test cannot reject the null hypothesis that points follow the same distribution across movements for Expert 2 (p=0.55). *Pairwise* comparisons sharpen these results, indicating significant distribution differences for two pairs out of ten (abstractism vs. cubism, abstractism vs. impressionism) for Expert 1 and three for Expert 3 (abstractism vs. cubism, cubism vs. surrealism, abstractism vs. expressionism). While significant differences could still happen in pairwise comparisons for Expert 2, the lowest *p*-value remains as high as 0.11 (cubism vs. impressionism).

*Quality ratings. Globally*, the same observations as for the RRMap ratings can be made, with similar *p*-values. *Pairwise*, significant differences can be noted for seven pairs for Expert 1, one pair for Expert 2 and six pairs for Expert 3, showing that for Experts 1 and 3, the movement often yields distribution differences in quality between AI-generated images and historical references.

*Takeaway.* Significant effects of movement can be noted for Experts 1 and 3 in both ratings distributions, especially present among some pairs of movements, while others are less distinguishable. Expert 2’s ratings might not be impacted by a particular movement.

#### 3.3.2. Effect of Criterion

*RRMap ratings. Globally*, all experts show a significant effect due to criterion in the ratings distributions. Given our nine criteria, there are 36 *pairwise* comparisons possible, among which 6 are significant for Expert 1, 9 for Expert 2, and 10 for Expert 3, with 3 pairs that are common for the three experts (painterly-photo. vs. top-bottom, left-right vs. painterly-photo., cent.-edges vs. parallel-inters.). We note, however, that this test relies on few sample sizes (15 points), making us remain cautious about the significance of these results.

*Quality ratings. Globally*, no significant differences are found in the quality ratings, i.e., we cannot reject the hypothesis that the distributions across the criteria are all the same. This is also reflected in the *pairwise* comparisons, where among the 36 possible ones, only 4 (resp. 0 and 1) are significant for Expert 1 (resp. 2, 3).

*Takeaway.* Significant effects of criterion can be noted for all experts in the RRMap ratings, globally and pairwise (among which 3 are common to all experts), while it is not (or less) the case for the quality ratings, which are thus likely more equally distributed across the criteria.

#### 3.3.3. Effect of Criterion Group

*RRMap ratings. Globally*, all the experts show significant effect of the three criterion group (topological, eidetic, chromatic) in the distributions. *Pairwise* comparisons show significant effects for 2 pairs out of 3 for Experts 1 and 3, and for one pair for Expert 2, which is common among the three experts (chromatic vs. topological). This could indicate an expert consensus on the difference in imitation capability of AI models with respect to chromatic and topological criteria.

*Quality ratings. Globally*, no expert shows a significant impact of criterion groups in quality proportions derived from the ratings. *Pairwise* comparisons show a similar trend with only 1 pair being significant, for Expert 1. This indicates that, when binned into quality categories as performed in this study, the potential distribution differences of the points in the RRMap tend to decrease, showing no signs of significant differences in quality.

*Takeaway.* Significant effects of criterion groups can be noted for all experts in the RRMap ratings, globally and pairwise (among which 1 is common to all experts), while it is not (or less) the case for the quality ratings, which are thus likely more equally distributed across the criteria groups.

#### 3.3.4. Effect of Modality

*RRMap ratings. Globally*, Expert 3 notes a significant effect of modality (movement, male painter, female painter) on the ratings distribution, backed up by two significant *pairwise* differences (females vs. movement, males vs. movement).

*Quality ratings.* The same observations can be made for quality ratings, with the addition of a significant difference for Expert 1 (females vs. movement).

*Takeaway.* There appear to be a few significant effects of modality in the experts ratings but no consensus. Interestingly, even though male painters are more famous and likely more present in training databases of text-to-image models, none of our experts noted a significantly better (or worse) reproduction of one gender over the other.

## 4. Discussion

### 4.1. Summary of Results

The present analyses reveal significant variability both across and within experts.

**Inter-expert variability.** Overall, the three experts produced significantly different distributions of both RRMap and quality ratings. Expert 3 was the least satisfied by AI-generated images (59% Bad, 32% Stereo), whereas Expert 2 was the most satisfied (61% Fair, 21% Excel.), and Expert 1 was in an intermediate position. These trends confirm strong expert effects. When examining options within categories (movement, criterion, criterion group, modality) separately, most rating distributions remained significantly distinct across experts, with only one possible agreement on the “sharp-blurry” criterion. Experts 1 and 2 showed the highest pairwise consistency, particularly in RRMap ratings and criterion-related quality ratings, while Expert 3 differed. Pointwise quality ratings further showed the limited consensus: only two points were identically rated by all experts, 43% of points fell into three different quality zones, and partial agreements were scattered across pairs. Overall, Experts 1 and 2 occasionally aligned on specific subsets of data (e.g., within a given criterion group and modality), yet no global consensus emerged.

**Intra-expert variability.** Category effects were then examined per expert. For the *movement* category, significant effects appeared for Experts 1 and 3, who differentiated several movement pairs (e.g., abstractism–cubism, cubism–surrealism), while Expert 2 showed more homogeneous ratings. The *criterion* category produced significant RRMap differences for all experts, with three common contrasts, although these effects were not observed in the quality ratings. Similarly, all experts showed significant RRMap differences across the three *criterion groups*, particularly between chromatic and topological criteria, yet no corresponding effect appeared in quality ratings. Finally, for the *modality* category, only Expert 3 showed consistent differences (female vs. movement, male vs. movement), with a weaker trend for Expert 1.

**Global takeaway.** These results tend to indicate (i) strong inter-expert divergence in both global and pairwise analyses; (ii) partial alignment between Experts 1 and 2, especially in RRMap-based evaluations; (iii) intra-expert effects of movements and criteria in Experts 1 and 3; and (iv) a recurring distinction between chromatic and topological criteria. When reduced to discrete quality levels, many of these fine-grained RRMap distinctions vanish. This might indicate that potential stylistic variations initially captured do not affect much the computed quality.

### 4.2. Additional Remarks and Limitations of the Results

It is worth noting that the statistical tests based on RRMap distributions operate directly on the raw ratings (up to a reversible normalization factor), whereas those derived from the discretized quality levels depend on the binning procedure and may thus introduce additional arbitrariness. Differences between RRMap- and quality-based outcomes should therefore be interpreted with caution, as they may depend on both methodological and perceptual factors. Moreover, our conclusions are inherently limited by the small number of experts involved. Expanding the panel to include a larger and more diverse group could yield more robust majority tendencies and clearer inter-expert agreements. Unfortunately, finding such experts and collecting their ratings is an extremely lengthy and difficult procedure. Finally, the historical (“real”) ratings obtained in this study can be viewed as defining a form of stylistic baseline (akin to a genotype characterizing each movement or painter) which could serve as a reference for future analyses comparing absolute stylistic values across generative models. We also anticipate that different text-to-image models, trained on distinct corpora and image–text pairings, might produce different stylistic distributions and therefore different outcomes under the same analytical protocol.

### 4.3. Closing the Loop: Expert Feedback

We started this research with a problem tied to art history, operationalized some criteria and used statistical analyses of experts ratings to handle it. To close the loop, we asked our experts their expected outcome before reading the results, and their opinion after reading the results.

Before reading the results, the comments indicate that the imitation of artistic movements and individual styles by generative AIs shows varying degrees of success depending on the observer’s level of demand. While the overall stylistic rendering often appears convincing, closer inspection reveals certain limitations. Concerning symmetry, for instance, Midjourney tends to accentuate left–right symmetry in compositions that are in fact predominantly asymmetrical. The treatment of blur also poses a problem: the AI produces vaporous effects with sharp contours, which fail to reproduce the instability characteristic of painterly blur. As for texture, a paradox emerges: brushstrokes are often more pronounced than in the original works, yet devoid of any real relief. Nevertheless, some plastic properties are generally well reproduced. Regarding the imitation of specific artists, it appears that the AI draws upon their canonical works within their overall production. Consequently, certain traits that belong to an artist but are not representative of the movement studied may occasionally appear. In short, the stylistic imitation is convincing at first glance, but closer examination reveals discrepancies.

After reading the article, the comments indicate that the statistical analyses corroborate initial impressions that stylistic imitation by AIs varies in quality across the evaluated criteria. The extent of inter-expert variability was deemed surprising, reminding us that any judgment remains partly subjective. In this context, the contribution of statistical analysis appears essential, as it helps to objectify certain impressions. The intuition that the overall rendering gives the illusion of conformity to a movement’s or artist’s stylistic traits, while closer scrutiny reveals deviations, finds an echo in the absence of significant effects of criterion groups on quality evaluations. The study demonstrates the value of combining qualitative expertise with quantitative validation to account for the complexity of stylistic judgment.

## 5. Conclusions

We proposed an interdisciplinary framework for assessing the stylistic fidelity of text-to-image generative models from an art-historical perspective. By combining expert ratings grounded in Greimas’s and Wölfflin’s visual theories with quantitative analyses of relative shifts, dispersions, and overlaps, we developed the *Relative Ratings Map* (RRMap) as an interpretable tool to compare ratings of historical artworks and generated images. Applied to Midjourney v6 across five major art movements and ten representative painters, our analyses revealed significant inter-expert variability but also meaningful intra-expert distinctions across movements and stylistic criteria. Overall, depending on the expert, the model succeeds more or less in imitating general stylistic tendencies, sometimes producing bad or stereotyped images, sometimes producing images faithful to historical artistic production according to the criteria used.

Furthermore, our overall framework can be used whenever one wants to assess the distribution difference between operationalized (on a continuous scale) rating characteristics of a reference image corpus and a query (for instance AI-generated) image corpus. Images do not need to be rated alone, as this is time-consuming and hardly consistent, which makes our framework practical: evaluations carried out “in bulk” reveal directly central tendencies and dispersions, and can be mapped to an RRMap for visual comparisons, even across different raters. Depending on the cases, the panel of raters might not need to be limited to experts, as basically anyone could be able to rate simple criteria, such as “darkness of hair” if images of faces need to be rated. The key really lies in the ability to operationalize rating criteria that can be understood and rated efficiently.

*Limitations and future work.* The observed expert divergences highlight the interpretive dimension inherent to style assessment and the need for larger, more diverse panels to consolidate future findings. In the same vein, studying more models could allow us to distinguish more significantly the inherent stylistic difficulties of text-to-image models from those that are model-specific. Beyond these empirical results, the framework itself offers a rigorous method for bridging qualitative expertise and quantitative rigor in the study of AI-generated art. Future work will extend this approach to additional models, expand the expert pool, and explore comparisons through time to examine how successive generative systems ingest and reproduce art history.

Beyond these extensions, we could foresee applications in various domains at the intersection of AI and artistic production. For instance, digital art curation could benefit from our framework to help distinguish between actual digital art (with known reference stylistic intents by the artists) and tentative imitations. Also, computational aesthetics could benefit from our framework, in the sense that experts in the field only need to define and operationalize their aesthetic criteria of interest before evaluating images and receive a RRMap quantifying the stylistic coherence and diversity of AI-produced images. Finally, the emerging topic of AI bias assessment could also be studied, by revealing systematic deviations and/or reductions in stylistic variability compared to a known (or expected) baseline of reference, thereby helping to identify and characterize stereotyped visual tendencies in generative models.

## Data Availability

The data presented in this study are openly available in FigShare at https://figshare.com/articles/dataset/Supplementary_material_JImaging-3990735/30695792?file=59803361 (accessed on 25 November 2025).

## Supplementary Materials

File S1: https://figshare.com/articles/dataset/Supplementary_material_JImaging-3990735/30695792?file=59803361 (accessed on 25 November 2025). It contains 660 MB of images (individual and stitched) generated for this study, as well as expert ratings.
